# Supplementary figures and images for: Accuracy of digital chest x-ray analysis with artificial intelligence software as a triage and screening tool in hospitalized patients being evaluated for tuberculosis in Lima, Peru
Source: medRxiv. 2023 Dec 7:2023.05.17.23290110. Originally published 2023 May 24. Preprint. [Version 2] doi: 10.1101/2023.05.17.23290110 (PMC10246158; doi:10.1101/2023.05.17.23290110)

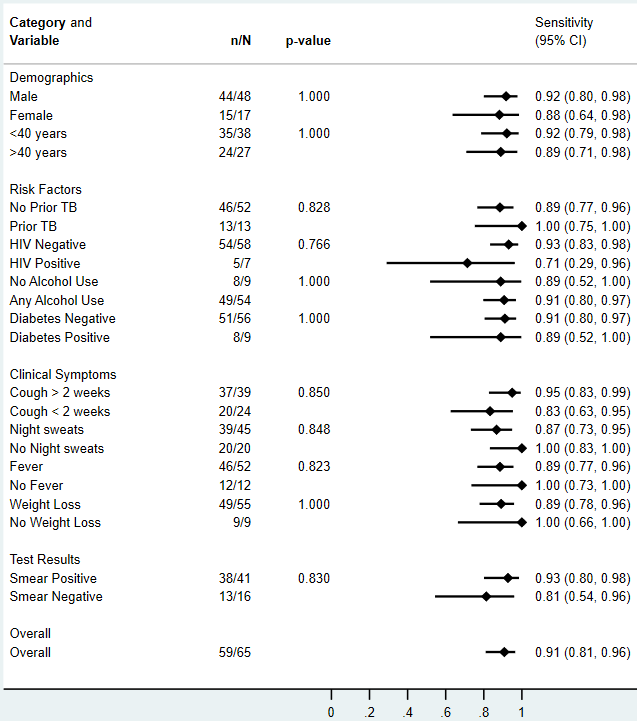

Supplement: Supplement 2 — Figure S1: Sensitivity of qXR version 3 for culture-confirmed pulmonary tuberculosis, overall and in prespecified stratified groups. p values are from Fisher’s exact tests. [file media-2.tif]

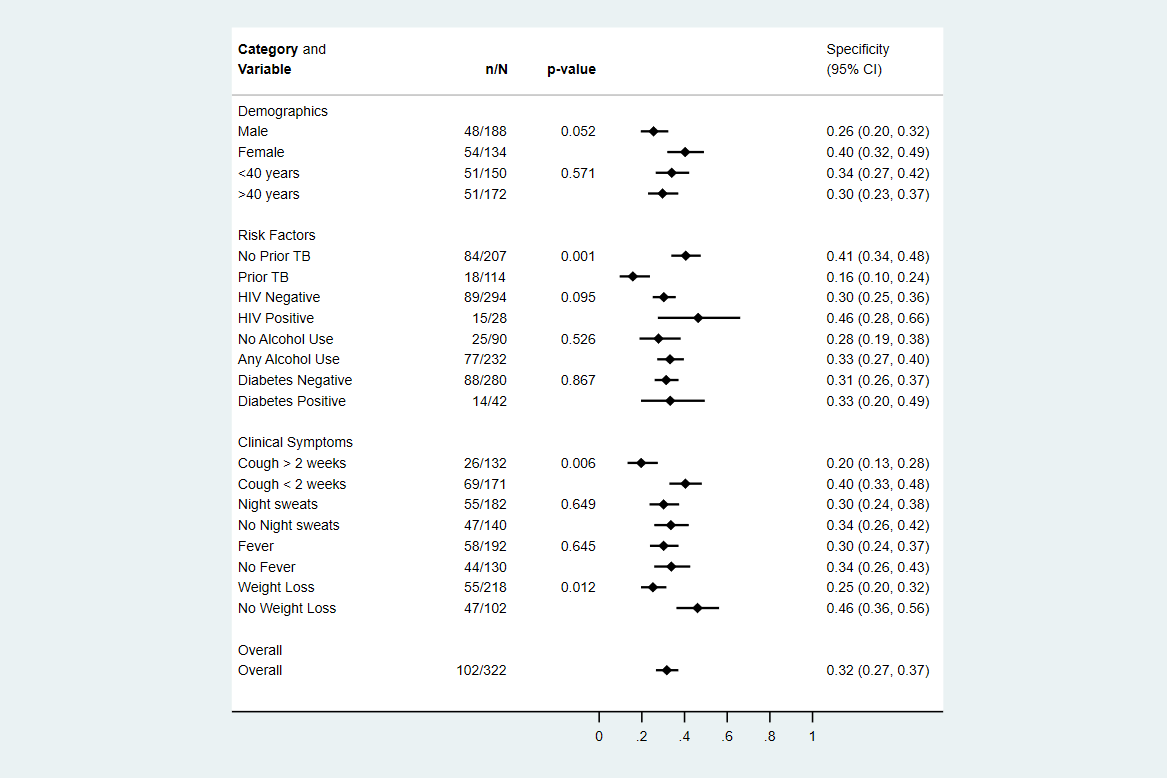

Supplement: Supplement 3 — Figure S2: Specificity of qXR version 3 for culture-confirmed pulmonary tuberculosis, overall and in prespecified stratified groups. p values are from Fisher’s exact tests. [file media-3.tif]
