# Supplementary material for: Accuracy of digital chest x-ray analysis with artificial intelligence software as a triage and screening tool in hospitalized patients being evaluated for tuberculosis in Lima, Peru
Source: medRxiv. 2023 May 24:2023.05.17.23290110. Preprint. [Version 1] doi: 10.1101/2023.05.17.23290110 (PMC10246158; doi:10.1101/2023.05.17.23290110)
Supplement: Supplementary file 1 [file NIHPP2023.05.17.23290110v1-supplement-1.pdf]

It is made available under a CC-BY 4.0 International license .

**Category** and **Variable**	**nN**	**p-value**	Specificity (95% CI)
Demographics			
Male	56/188	0.065	0.30 (0.23, 0.37)
Female	60/134		0.45 (0.36, 0.54)
<40 years	59/150	0.449	0.39 (0.32, 0.48)
>40 years	57/172		0.33 (0.26, 0.41)
Risk Factors			
No Prior TB	89/207	0.015	0.43 (0.36, 0.50)
Prior TB	27/114		0.24 (0.16, 0.33)
HIV Negative	101/294	0.204	0.34 (0.29, 0.40)
HIV Positive	13/22		0.54 (0.34, 0.72)
Clinical Symptoms			
Cough > 2 weeks	26/132	0.001	0.20 (0.13, 0.28)
Cough < 2 weeks	79/171		0.46 (0.39, 0.54)
Night sweats	62/182	0.587	0.34 (0.27, 0.41)
No Night sweats	54/140		0.39 (0.30, 0.47)
Fever	63/192	0.325	0.33 (0.26, 0.40)
No Fever	53/130		0.41 (0.32, 0.50)
Weight Loss	68/218	0.069	0.31 (0.25, 0.38)
No Weight Loss	48/102		0.47 (0.37, 0.57)
Overall			
Overall	116/322		0.36 (0.31, 0.41)

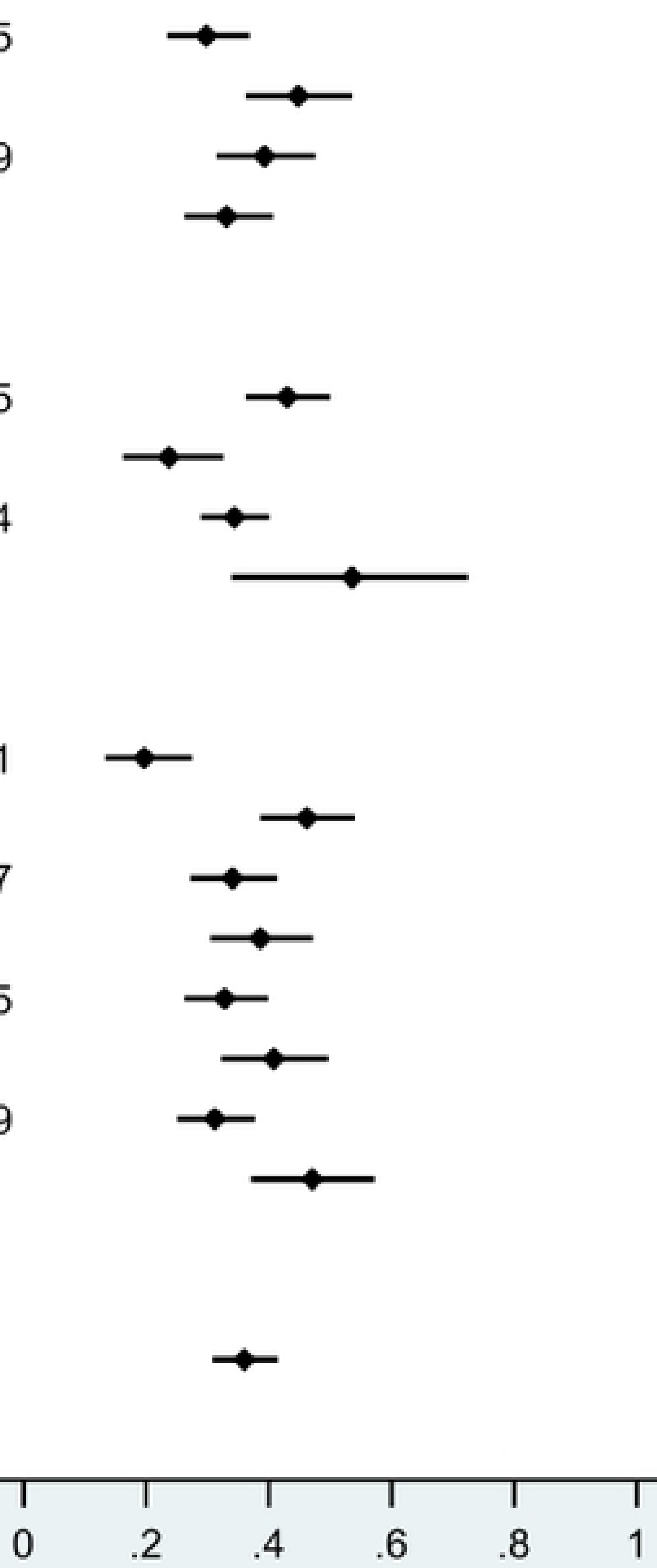

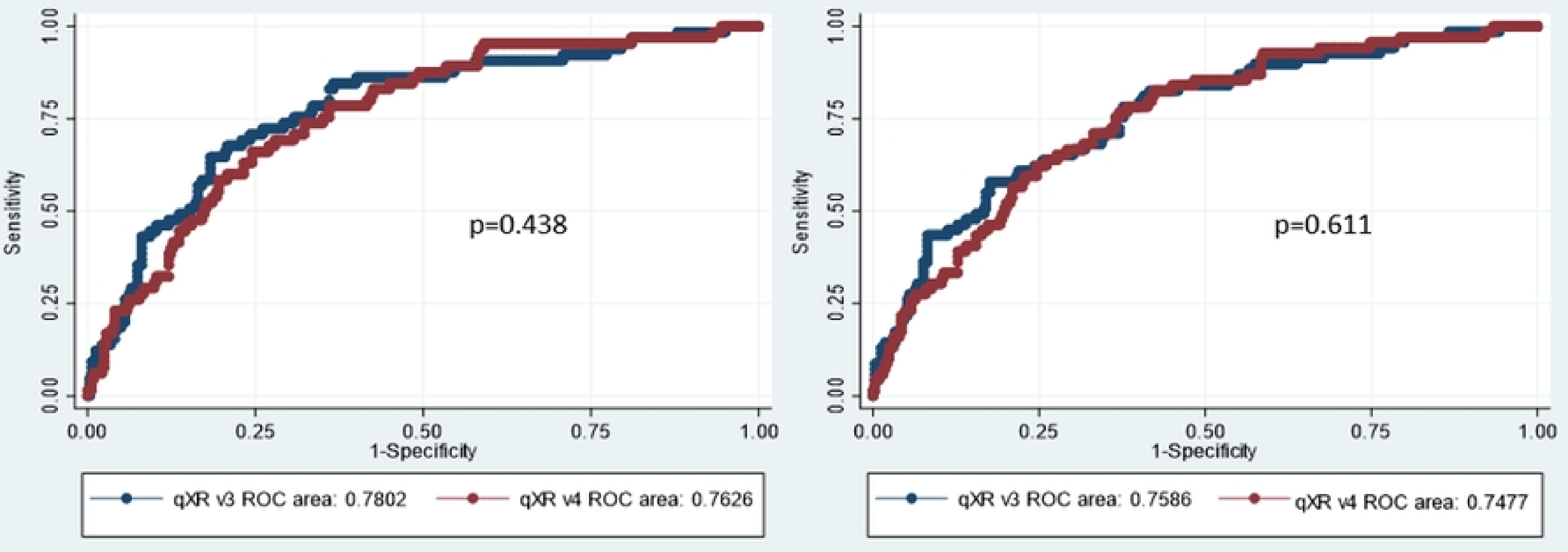
Sensitivity
1.00
0.75
0.50
0.25
0.00
0.00
0.25
0.50
0.75
1.00
1-Specificity
p=0.438
qXR v3 ROC area: 0.7802
qXR v4 ROC area: 0.7626
Sensitivity
1.00
0.75
0.50
0.25
0.00
0.00
0.25
0.50
0.75
1.00
1-Specificity
p=0.611
qXR v3 ROC area: 0.7586
qXR v4 ROC area: 0.7477
